# Evidence of involvement of the mannose receptor in the internalization of *Streptococcus pneumoniae* by Schwann cells

**DOI:** 10.1186/s12866-014-0211-9

**Published:** 2014-08-02

**Authors:** Hugo Macedo-Ramos, Andre F Batista, Alvaro Carrier-Ruiz, Lucineia Alves, Silvana Allodi, Victor T Ribeiro-Resende, Lucia M Teixeira, Wagner Baetas-da-Cruz

**Affiliations:** 1 Faculdade de Medicina, Centro de Cirurgia Experimental, Laboratório Translacional em Fisiologia Molecular, Universidade Federal do Rio de Janeiro, Rio de Janeiro, RJ, Brazil; 2 Instituto de Biofísica Carlos Chagas Filho, Laboratório de Neurobiologia Comparativa e do Desenvolvimento, Universidade Federal do Rio de Janeiro, Rio de Janeiro, RJ, Brazil; 3 Instituto de Microbiologia Paulo de Góes, Universidade Federal do Rio de Janeiro, Rio de Janeiro, RJ, Brazil; 4 Instituto de Biofísica Carlos Chagas Filho - Pólo de Xerém, Laboratório de Neuroquímica, Universidade Federal do Rio de Janeiro, Rio de Janeiro, RJ, Brazil; 5 Instituto de Biofísica Carlos Chagas Filho, Laboratório de Neurobiologia Celular e Molecular, Universidade Federal do Rio de Janeiro, Rio de Janeiro, RJ, Brazil; 6 Instituto de Biofísica Carlos Chagas Filho, Programa de Pós-Graduação em Ciências Biológicas (Fisiologia), Universidade Federal do Rio de Janeiro, Rio de Janeiro, RJ, Brazil

**Keywords:** Pneumococcal meningitis, Glia, Pattern recognition receptor (PRRs), Innate immunity, Pathogen-associated molecular patterns (PAMPs), Nerve injury

## Abstract

**Background:**

The ability of *S. pneumoniae* to generate infections depends on the restrictions imposed by the host’s immunity, in order to prevent the bacterium from spreading from the nasopharynx to other tissues, such as the brain. Some authors claim that strains of *S. pneumoniae*, which fail to survive in the bloodstream, can enter the brain directly from the nasal cavity by axonal transport through the olfactory and/or trigeminal nerves. However, from the immunological point of view, glial cells are far more responsive to bacterial infections than are neurons. This hypothesis is consistent with several recent reports showing that bacteria can infect glial cells from the olfactory bulb and trigeminal ganglia. Since our group previously demonstrated that Schwann cells (SCs) express a functional and appropriately regulated mannose receptor (MR), we decided to test whether SCs are involved in the internalization of *S. pneumoniae* via MR.

**Results:**

Immediately after the interaction step, as well as 3 h later, the percentage of association was approximately 56.5%, decreasing to 47.2% and 40.8% after 12 and 24 h, respectively. Competition assays by adding a 100-fold excess of mannan prior to the *S. pneumoniae* infection reduced the number of infected cells at 3 and 24 h. A cytochemistry assay with Man/BSA-FITC binding was performed in order to verify a possible overlap between mannosylated ligands and internalized bacteria. Incubation of the SCs with Man/BSA-FITC resulted in a large number of intracellular *S. pneumoniae,* with nearly complete loss of the capsule. Moreover, the anti-pneumococcal antiserum staining colocalized with the internalized man/BSA-FITC, suggesting that both markers are present within the same endocytic compartment of the SC.

**Conclusions:**

Our data offer novel evidence that SCs could be essential for pneumococcal cells to escape phagocytosis and killing by innate immune cells. On the other hand, the results also support the idea that SCs are immunocompetent cells of the PNS that can mediate an efficient immune response against pathogens via MR.

## Background

*Streptococcus pneumoniae* is a Gram-positive bacterial pathogen that commonly colonizes the human respiratory tract. The ability of *S. pneumoniae* to generate infections depends on the restrictions imposed by the host’s immunity, in order to prevent its spread from the nasopharynx to other tissues and sites, such as the middle ear, lungs, blood, and brain [1]. The means by which some strains of *S. pneumoniae* invade the brain without the occurrence of bacteremia are still unknown. Some authors claim that strains of *S. pneumoniae*, failing to survive in the bloodstream, can enter the Central Nervous System (CNS) directly from the nasal cavity by axonal transport through the olfactory nerves or trigeminal ganglia [2]. However, from the immunological point of view, glial cells are far more responsive to bacterial infections than are neurons, and therefore more likely to internalize them. This hypothesis is consistent with several recent reports showing that bacteria can infect glial cells from the olfactory bulb and trigeminal ganglia, such as Olfactory Ensheathing Cells (OECs) and Schwann cells (SCs), respectively [3]–[5].

SCs are glial cells that are closely associated with the peripheral nerves, and can be classified into two types: myelinating and non-myelinating. Myelinating Schwann cells provide the myelin sheath of individual axons, and non-myelinating Schwann cells ensheathe several small axons. Both SC phenotypes can interface with the external environment through nerve endings scattered in the mucosa, and thus can potentially interact with pathogens. Several lines of evidence suggest that SCs can function as sentinel cells in the peripheral nervous system (PNS), and are a potent source of cytokines and innate immune receptors (pattern recognition receptors [PRRs]), such as Toll-like receptors (TLRs) and Mannose Receptors (MR), which are capable of controlling adaptive immune responses against self- and non-self antigens [6]–[9].

MR is a 175-kDa transmembrane glycoprotein receptor that contains multiple domains in the extracellular region, including Ca^2+^-dependent lectin-like carbohydrate recognition (CTLD), responsible for the binding to mannose, fucose, and N-acetylglucosamine, present in small molecular motifs called pathogen-associated molecular patterns (PAMPs) and damage-associated molecular patterns (DAMPs) [10]–[12]. MR has emerged as an important component of the innate immune system, participating in host defense following microbial infections. This receptor can initiate host mechanisms to remove pathogens, most specifically through activated macrophages. However, other cell types express MR in a functional state able to recognize and internalize microbial components [13]. MR is involved in the innate immune response in several tissues [14],[15], including normal and injured nerve tissue, where it was found to express in microglia, astrocytes, immature neurons, Schwann cells, and olfactory ensheathing cells [16],[7],[3],[17],[18]. However, there is no evidence that either mature oligodendrocytes or their precursors express MR [19].

By using different models of interaction with some highly mannosylated ligands, our group previously demonstrated that SCs express a functional and appropriately regulated MR [20],[7]. We also demonstrated that SCs may harbor infectious agents and act as safe hosts by producing immune mediators [21],[22]. In the present study, we evaluated whether SCs cultured from the adult sciatic nerve are able to internalize *S. pneumoniae* via RM.

## Methods

### Animals

One-month-old Wistar rats were used to obtain primary SC cultures. Animal care and euthanasia procedures followed the norms established by the Brazilian Society for Neuroscience (SBNeC), as well as by the ethics committees of the Institute of Biophysics Carlos Chagas Filho of the Federal University of Rio de Janeiro (IBCCF/UFRJ - Permit Number: 158).

### Schwann cell cultures

Primary rat SCs were obtained according to a modification by P.M. Wood of the procedure described by Morrissey et al. [23]. Briefly, sciatic nerves were harvested in Leibovitz’s L 15 Medium (Invitrogen, Carlsbad, CA, USA), fragmented, and cultured in Dulbecco’s Modified Eagle’s Medium (DMEM; Invitrogen) containing 10% heat-inactivated fetal calf serum (FCS; Cultilab, Campinas, Brazil).

After 30 days, the nerve fragments were treated with 0.5 mg/ml collagenase type I (Worthington Biochemicals, New Jersey, NJ, USA) and 1 mg/ml dispase II (Roche Molecular Biochemicals, Indianapolis, IN, USA) overnight in a CO_2_ incubator at 37°C for dissociation. After washing, the cells were cultured in dishes covered with a solution containing poly-L-lysine (200 μg/ml, Sigma Chemical, St. Louis, MO, USA), in DMEM containing 10% FCS, 100 U/ml penicillin (Invitrogen), 100 μg/ml streptomycin (Invitrogen), 2 μM forskolin (Calbiochem, La Jolla, CA, USA), and 20 μg/ml bovine pituitary extract (Biomedical Technologies, Stoughton, MA, USA). After the first passage, SCs were further selected from fibroblasts by using an anti-mouse Thy 1.1 antibody (undiluted hybridoma culture supernatant, American Tissue Culture Collection, Manassas, VA, USA) and rabbit complement (Sigma). This resulted in approximately 97 - 99% pure SC cultures as assessed by S100-β (DAKO, Carpinteria, CA, USA) immunoreactivity. SC-enriched cultures were maintained in a humidified air/CO_2_ (95%/5%) atmosphere at 37°C.

Because a limited amount of primary SCs was available, pilot experiments were performed with the ST88-14 tumor cell line (Schwannoma cells). The ST88-14 cells, isolated from a patient with neurofibromatosis type 1 [24], were kindly donated by J.A. Flechter (Dana-Farber Cancer Institute, Boston, MA, USA). For inclusion in the present study, the cells were grown in RPMI 1640 medium supplemented with 5% FCS, 1 mM glutamine, 1000 U/ml penicillin, and 50 μg/ml streptomycin. All chemicals were from Sigma. The cells, plated in culture dishes or on cover slips in 24-well plates (Falcon, Franklin Lakes, NJ, USA), were maintained in a humidified air/CO_2_ (95%/5%) atmosphere at 37°C for 24 h.

### Phenotypic identification of SCs

The SCs cultures, both ST88-14 cells and Schwann cell primary cultures, were treated with PBS + 0.3% Triton X-100 (Sigma) and blocked with 10% normal goat serum (NGS). For phenotypic identification of SCs, the cultures were incubated with mouse monoclonal antibody anti-S100-β (Sigma), a Schwann cell marker [25]. After reaction with the primary antibodies of interest, cells were incubated with goat anti-rabbit IgG and/or goat anti-mouse IgG secondary antibodies. Soon after, the cells were washed in PBS pH 7.4 and mounted with N-propylgallate in PBS-glycerol and coverslipped.

### Expression of MR and uptake of a mannosylated neoglycoprotein by SCs

SCs were tested for the expression of MR by labeling with a polyclonal antibody, produced in rabbits, directed against a C-terminal peptide of murine MR (anti-cMR, 1/100), kindly donated by Dr. Anne Régnier-Vigouroux [19]. A cytochemistry assay with 50 μg/ml of the neoglycoprotein mannosyl/bovine serum albumin-FITC-conjugated (man/BSA-FITC, Sigma) diluted in Ringer solution containing 5 mM CaCl_2_ and 1% BSA at 37°C for 1 h was performed in order to confirm the internalization pattern in SCs. Both expression and functional analyses (MR-mediated endocytosis) of the MR in SCs were performed as previously described by us in detail [20],[7].

### Interaction assay of *S. pneumoniae* and SCs

Strain *S. pneumoniae* ATCC 49619 (American Type Culture Collection 49619) was selected for performing interaction assays with SCs, based on the facts that (1) it is a reference strain widely used in medical microbiology research and diagnostic laboratories worldwide; and (2) it belongs to serotype 19 F, which is frequently associated with pneumococcal infections in many parts of the world and is often linked to resistance to penicillin and other antimicrobial agents [26].

For interaction assays, bacterial cells were obtained by streaking strain ATCC 49619 on 5% sheep blood agar plates (Plast Labor, Rio de Janeiro, RJ, Brazil). After incubation at 37°C for 20 h under 5% CO_2_ atmosphere, individual colonies were selected and cells were suspended in Hanks' balanced salt solution (HBSS; Sigma) to reach a turbidity equivalent to the 0.5 McFarland standard. To reduce cell clumping, the bacterial suspension was passed 15 times through a 27-gauge needle and then allowed to settle for 15 min. Only the top fraction of the suspension containing dispersed bacteria was used to infect SCs. This dissociation method was used only in the case of bacterial clumping.

First, we determined the number of SCs using a Neubauer Chamber. Next, the bacterial inoculum was determined by McFarland Turbidity Standards. SC cultures were infected with suspensions of living *S. pneumoniae* ATCC 49619 cells in a ratio of 100:1 bacteria/SC cells for at least 3 h in serum- and antibiotic-free DMEM F-12. After this period, the cultures were rinsed with PBS to remove non-adhered bacteria, DMEM F-12 was added, and the infection was followed at 37°C for up to 24 h, with fixation of infected cells at 3, 12, and 24 h after PBS rinsing. The number of SCs associated with *S. pneumoniae* was determined after 3, 12 and 24 h.

For the dark-field microscopy analyses, the infected and uninfected cultures were washed in PBS and fixed. The samples on cover slips, previously fixed in 4% paraformaldehyde at room temperature, were permeabilized with PBS-Triton 0.3% and blocked with 10% NGS [27],[3]. After that, bacteria were detected by using a Pneumococcal anti-serum (OMNI States Serum Institut, Copenhagen, Denmark) and/or stained with 0.1 mg/ml 4’,6-diamidino-phenylindole (DAPI, Sigma). The viability of the bacteria was examined using fluorescent microscopy after staining with 5 mM SYTOX Green nucleic acid stain (Invitrogen) [28].

Competition assays were performed by infecting cultures in the presence of 100 μg/ml of mannan (hyper-mannosylated glycoprotein from *Saccharomyces cerevisiae* - Sigma) after testing concentrations in the range of 10 to 1000 μg/ml (10, 100, 500, and 1000 μg/ml) [29],[30],[3] for 3 to 24 h. A cytochemical assay with (Man/BSA-FITC) binding was performed in order to determine the presence of a MR with the active CTLDs. Other infected cultures were incubated with 50 μg/ml man/BSA-FITC as described above. The glass coverslips were then removed from each well and mounted on glass slides with entellan glue plus 90% glycerol in PBS containing 1 μg/ml p-phenylenediamine [27]. Samples were analyzed using a Zeiss epifluorescence photomicroscope (Zeiss, Jena, Germany) and a set of 200 cells was examined for the presence of *S. pneumoniae*. In addition, the percentage of cells with associated bacteria (adhered or internalized) was calculated as follows: number of infected cells/200 cells × 100.

### Confocal microscopy

Cells were seeded at a density of 1.2 × 10^6^ cells/ml in DMEM F-12 medium plus 10% FCS on poly-L-lysine plus laminin-coated glass coverslips for 30 min at 37°C and mounted in N-propylgallate (Sigma) in PBS-glycerol. The samples were placed under a Leica TCS SP5 confocal microscope (Leica Microsystems, Heidelberg, Germany) and all images were acquired with a 63X glycerol immersion objective lens. Image treatment was performed using the Image Processing Leica Confocal and ImageJ Software (Wayne Rasband, National Institutes of Health, Bethesda, MD, USA). The three-dimensional sections perpendicular to the plane of the monolayer and parallel to the x or y axis were reconstructed using Leica Application Suite Advanced Fluorescence (LAS AF) software.

### Statistical analysis

Statistical analyses of the data from assays of competition and of cell/bacteria association were performed with One-way ANOVA followed by the Tukey test for multiple comparisons. In case of single comparisons, the Student *t* test was applied. *P* values equal to or less than *0.05* were considered statistically significant.

## Results and discussion

The present study is focused on the interaction between *S. pneumoniae*, a major agent of bacterial meningitis, and glial cells, which are currently considered as part of the innate immune system, forming a first line of defense against infections of the nervous system. We used a model of infection of glial cells by *S. pneumoniae*. This model was improved during previous studies by our group, which showed that the bacterial load and time course of infection are crucial in this *in vitro* model [3].

Recent studies have shown that glial cells are highly reactive to pathogens, through regulating inflammation, and participating in innate and adaptive immunity [5],[31]–[34]. In the specific case of SCs, it has been shown that, similarly to microglia in the brain, they may act as sentinel cells in the PNS and thus orchestrate the induction of a host defense response [35],[8]. Recent data from our group indicate that SCs from the rat sciatic nerve and a human SC line (ST88-14) express MR in a functional state capable of internalizing mannosylated ligand [20],[7]. We also have previously shown that cells egress from sciatic nerve explant cultures treated with IFN-γ, MHC class II staining colocalized with internalized neoglycoprotein in perinuclear areas of cells phenotypically identified as SC [7]. These findings are consistent with a possible role of SC in the clearance of DAMPs and PAMPs, acting as facultative antigen-presenting cells during inflammation. Furthermore, we have previously demonstrated that a human SC line (ST88-14 cells) is able to internalize promastigote forms of *Leishmania amazonensis* and that, in subsequent steps to infection, the parasite triggers cellular signal transduction pathways, inducing the nuclear translocation of the nuclear factor-kappa B (NF-kappa B) [21].

**Figure 1:**
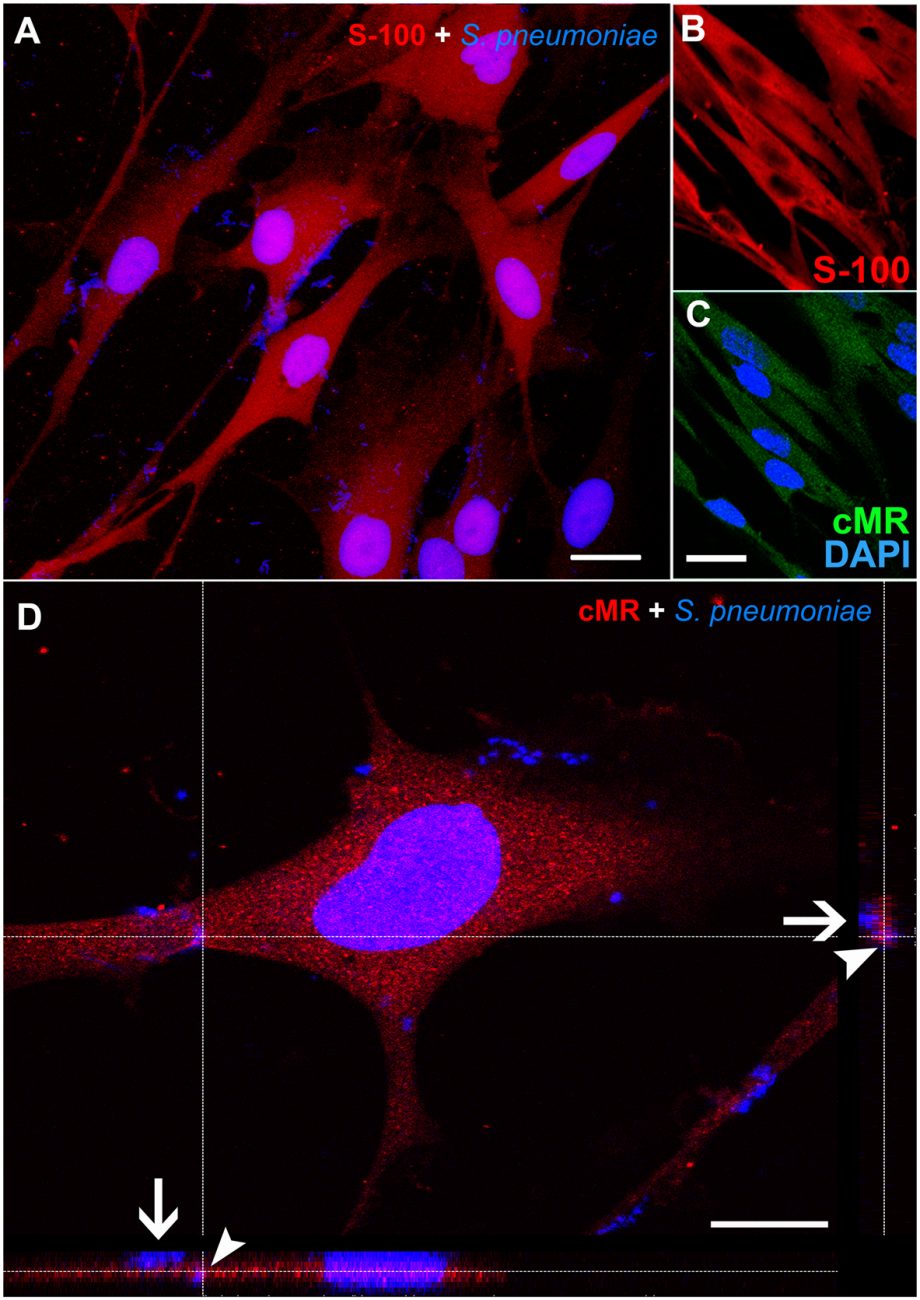
**Confocal microscopy images showing expression of the mannose receptor (MR) in uninfected and infected Schwann cells (SCs) by*Streptococcus pneumoniae*. (A)** Optical sections showing the expression of S100-β in infected Schwann cells (SCs) cultured from the adult sciatic nerve. **(B and C)** Double immunolabeled images, showing in **B**, uninfected SCs labeled for S100-β in red (maximum nuclear diameter), and in **C**, the same cells immunolabeled for the mannose receptor (cMR) conjugated with Alexa Fluor 488. The same field demonstrates that all S100-β^+^ cells are also labeled with cMR (plane at or near the free surface of the membrane). **(D)** Optical section, where SCs infected by *S. pneumoniae* for 3 h were immunolabeled for cMR (red). Bacteria were stained with DAPI (blue). Orthogonal z-sections in the horizontal and vertical planes reveal *S. pneumoniae* adhered (arrow) or internalized (arrowheads) by SCs **(D)**. The nuclei were counterstained with DAPI. These results are representative of five separate experiments. Scale bar = 18 μm in **(A)**; 18 μm in **(B - C)**; 12 μm in **(D)**.

The ST88-14 SC line is a good model for the present study because these cells express some phenotypic markers of normal SCs [36]. In view of this and because a limited amount of primary SCs and an overwhelming quantity of ST88-14 cells were available, the pilot experiments were performed with ST88-14 cells. After standardization of the protocols, the same tests were repeated with primary SCs. No significant differences were observed between the two cell types in any of the experiments. To confirm the Schwann-like nature of our ST88-14 cells and the purity of the SC preparation obtained from primary cultures, both cultures were incubated with polyclonal anti-S100-β antibody. All or virtually all ST88-14 cells showed marked positivity for S100β protein (not shown). Correlative microscopy of images obtained in phase-contrast and confocal immunofluorescence optics showed S100-β^+^ cells, and revealed a high degree of purity in our primary SC cultures (Figure 1B). The purity of isolated primary SCs exceeded 97 - 99%, as previously described by our group [7]. Incubation of fixed SCs with the cMR antibody resulted in distinct labeling, widely distributed both on the surface and in the cytoplasmic domain (different optic planes selected from z-series) of SC from primary nerve cultures (Figure 1C), confirming our previous data [7]. Omission of the primary antibodies eliminated the respective labeling (not shown). In an initial approach, we evaluated whether SCs could harbor *S. pneumoniae* in an *in vitro* model of infection. Our results revealed a variable number of internalized bacteria throughout the cytoplasm of SCs (Figure 1A). To confirm that the MR was involved in the uptake of *S. pneumoniae*, SCs were reacted with anti-cMR. In order to solve the problem caused by the use of two antibodies produced in rabbits, the bacteria were revealed with DAPI. These results showed an intense immunoreaction with anti-cMR in intracellular compartments containing *S. pneumoniae* (Figure 1D) of SCs previously identified by the anti-S100-β antibody (Figure 1A).

**Figure 2:**
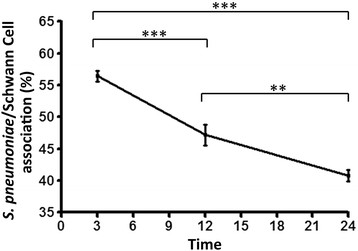
**Kinetics of association (adhesion or internalization) of*Streptococcus pneumoniae*with Schwann cells (SCs).** The percentage of SCs containing adhered or internalized *S. pneumoniae* was quantified at different times up to 24 h. The graph shows a progressive decrease in the number of *S. pneumoniae* associated with the SCs. These data are representative of three separate experiments, each of which was conducted in triplicate. ***P <0.0001. For statistical analysis, we used Two-way ANOVA and Tukey’s Multiple Comparison Test.

To monitor the course of infection, the number of SCs containing adhered and/or internalized *S. pneumoniae* was quantified at different times up to 24 h. Immediately after the interaction step, as well as 3 h later, the percentage of association was 56.5%, and decreased to 47.2% and 40.8% after 12 and 24 h, respectively (Figure 2).

**Figure 3:**
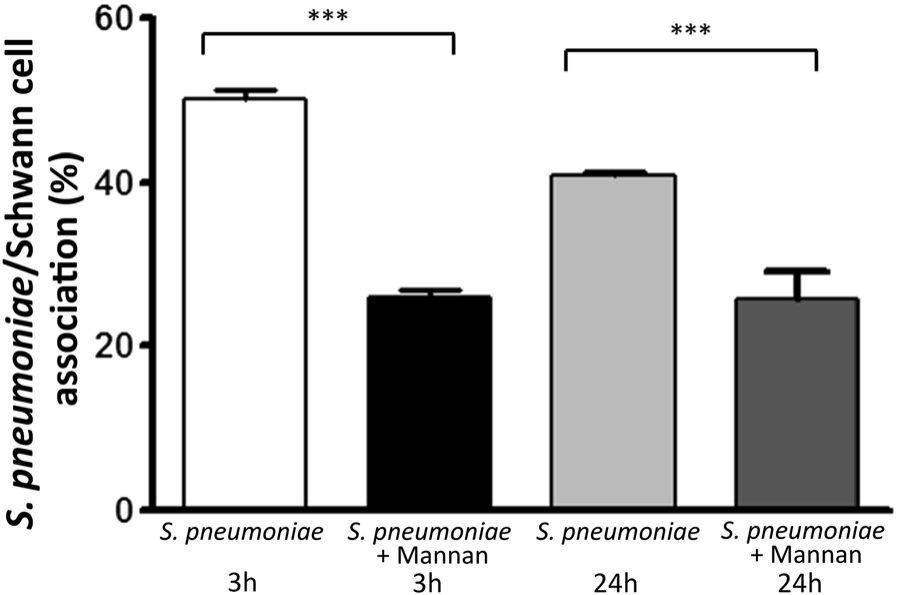
**Competition assays showing the participation of mannose receptor (MR) during the association of*Streptococcus pneumoniae*with Schwann cells (SCs).** The assays were performed by adding increasing doses of mannan (10 to 1000 μg/ml) in the interaction medium, and the results were highly statistically significant (***P <0.0001) at a dose equal to or higher than 100 μg/ml. The graph shows an inhibition of the percentage of SCs with associated bacteria immediately after 3 h of association (black bar versus white bar). However, this percentage was not significantly affected after this time up to 24 h of infection in mannan-treated cultures (black bar versus dark-gray bar). These data are representative of three separate experiments, each of which was conducted in triplicate. For statistical analysis, we used Two-way ANOVA and Tukey’s Multiple Comparison Test.

**Figure 4:**
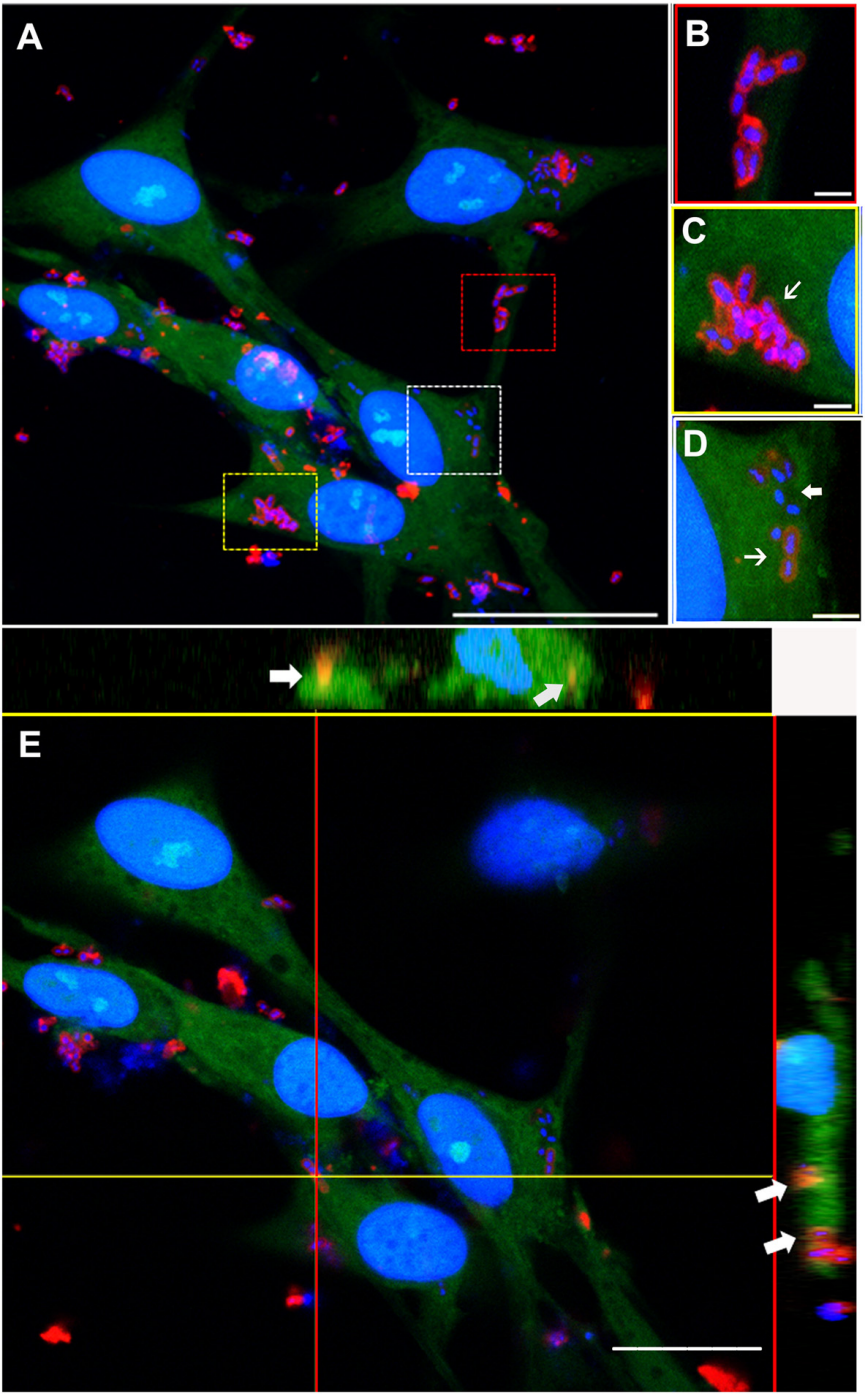
**Confocal microscopy analysis of the mannosyl/bovine serum albumin-fluorescein isothiocyanate (man/BSA-FITC) colocalization with*Streptococcus pneumoniae*capsule in Schwann cells (SC). (A)** Optical section of infected Schwann cells cultured for 48 h, immunolabeled for anti-pneumococcal antiserum (red) and reacted with Man/BSA-FITC (green). Active CTLDs of MR in infected SCs were observed after receptor-ligand binding assays with Man/BSA-FITC (red, yellow and white dashed squares in A). Higher-magnification views of the red, yellow and white boxes in A show details of *S. pneumoniae* adhered to the cellular surface **(B)** or internalized by SC in **C** and **D**. Internalized bacteria can be seen throughout the cytoplasm of the SCs (thin arrows in **C** and **D**), some of which lack the polysaccharide capsule (thick arrow in **D**). **(E)** Optical section at the maximum nuclei diameter of **A** with the orthogonal plane images cut at the yellow and red lines, and projected in the upper and right columns, respectively. Orthogonal projections show colocalization of both markers (arrows). The nuclei of SCs and/or bacterial DNA (blue dots) are stained with DAPI. The DAPI counterstaining shows the bacterial DNA surrounded by intense labeling of the pneumococcus capsule that reacted with the anti-pneumococcal antiserum **(B - D)**. These results are representative of five separate experiments. Scale bar = 30 μm in **(A)**; 1.5 μm in **(B)**; 2 μm in **(C - D)**; 18 μm in **(E)**.

We evaluated the endocytosis of *S. pneumoniae* by SCs, maintained either in medium alone or in medium containing an excess of mannan, according to a protocol previously described by us for the endocytosis of *S. pneumoniae* by OECs [3]. Observations were made after interaction of *S. pneumoniae* with SCs for 3, 12, and 24 h in both conditions. Variable numbers of internalized bacteria as detected by labeling with anti-pneumococcal antiserum and counterstained with DAPI were seen throughout the cytoplasm of SCs maintained in medium alone (Figure 3, detailed in Figure 4A-E). On the other hand, the interaction assays performed in the presence of mannan impaired the bacterial binding to the cellular surfaces, thus drastically reducing the number of infected cells after 3 h of association (Figure 3). However, the number of infected cells was not significantly affected from 3 to 24 h of infection in the mannan-treated cultures (Figure 3).

The results of the present study suggest that MR is involved in infection of SCs by *S. pneumoniae* in a specific manner. Competition assays conducted by adding a 100-fold excess of mannan prior to the infection with *S. pneumoniae*, confirmed the participation of MR during the association of bacteria with SCs. This result suggests the presence of a receptor-ligand recognition system employed by *S. pneumoniae* for invasion of the SCs, since incubation of the cell cultures with latex beads 2 μm in diameter (non-mannosylated particle) did not result in a change in the number of infected SCs (not shown).

The reduction in the percentage of infected SCs after 12 and 24 h of association can also be attributed to a phenomenon known as pneumococcal fratricide, which causes the activation of LytA to disrupt completely the cell wall of noncompetent bacteria. [37]–[39]. We hypothesized that this fratricide phenomenon may also explain why no differences were found between 3 and 24 h of infection in mannan-treated cultures, since competition of bacteria/mannan for binding sites on the cell surface may have selected bacteria with different abilities to cause infection prior to saturation of these sites. Similar results were obtained in our previous studies on the interaction of OECs with *S. pneumoniae,* indicating the presence of a functional MR expressed on the OECs cellular surface, which binds the capsule from bacteria in a mannan-inhibitable manner [3].

Previous studies using animal models have shown that the capsular polysaccharide might influence the proportion of bacteria capable of adhering to and invading the cells [40]. Other studies suggest that polysaccharide conformation may play an important role in pneumococcal recognition [13]. Additionally, the MR was found to bind to purified capsular polysaccharides of *S. pneumoniae* and to the lipopolysaccharides, but not capsular polysaccharides, of *Klebsiella pneumoniae*. However, no direct correlation can be made between polysaccharide structures and recognition by MR, since, although they were Ca^2+^-dependent and inhibitable by D-mannose, these polysaccharides had none of the structural features often associated with known MR [13]. It may be possible that *S. pneumoniae* changes some capsular structures after an initial contact of their mannosylated residues with the MR of the host cell surface, and hence may also interact with other non-lectin domains of the receptor.

The morphology of the bacteria was analyzed by confocal microscopy. As might be expected, adhered bacteria were easily recognized by their uniform size, smooth contour, and neat arrangement in diplococcus-shaped pairs, similar to the appearance commonly observed in bacterial cultures. There were no significant morphological changes in the extracellular bacteria before or after the experiments.

Cytochemistry assays with Man/BSA-FITC binding were performed in order to verify a possible colocalization between a mannosylated ligand and internalized *S. pneumoniae*. Similarly to the report in our previous studies [20],[7], incubation of uninfected SCs with Man/BSA-FITC showed an intense labeling, widely distributed on the cellular surface and also in the intracellular domain. However, this pattern was not significantly affected by bacterial infection. For negative controls, the same Man/BSA-FITC reactions performed in the presence of 250 mM D-mannose resulted in loss of the Man/BSA-FITC labeling in SC tagged by anti-S100-β antibody (not shown). *S. pneumoniae* was localized predominantly in cytoplasmic compartments, with intense staining for Man/BSA-FITC, presumably defining edges of the vesicles (Figure 4A, C and D). Only small numbers of *S. pneumoniae* were bound to the SC surface (Figure 4B). Moreover, the anti-pneumococcal antiserum staining colocalized with the internalized man/BSA-FITC, suggesting that both markers are present within the same endocytic compartment of the SC (Figure 4E).

Interestingly, incubation of the SCs with Man/BSA-FITC resulted in a large number of intracellular *S. pneumoniae* cells with a nearly complete loss of the capsule (Figure 4D). In addition, large numbers of *S. pneumoniae* internalized by SC in a nonencapsulated form were observed after 3 h of infection, but no substantial loss of bacterial viability was observed under these conditions after washing and recovery of living bacteria from the lysed cell host. Nevertheless, we cannot rule out this possibility, since previous studies showed that during alveolar macrophage infection, significantly more intracellular nonencapsulated *S. pneumoniae* were killed than the capsulated form [41]. In fact, we observed a reduction in the number of infected cells immediately after 3 h of association of *S. pneumoniae* with SCs followed at different times up to 24 h. Several aspects may be associated with this finding, including the ability of bacteria to escape from endocytic vesicles and then migrate to the extracellular environment [42], or die, either immediately after the adhesion or during internalization [39]. However, continued studies are necessary to better understand this mechanism in our model.

## Conclusions

Our study provided new insights into the molecular and cellular mechanisms by which *S. pneumoniae* can gain access to the CNS in the absence of bacteremia. The nasopharynx and maxillary sinuses are richly innervated by myelinated and non-myelinated sensory axons (and their associated Schwann cells) from the trigeminal nerve; thus, it can be predicted that any infection of SCs in these regions could provide a means of transport for *S. pneumoniae* toward the brain along the peripheral nerves. Moreover, considering that *S. pneumoniae* is a common commensal in the nasopharynx of healthy adults and children, any surgical procedure in this region could result in a risk of contamination. Actually, pneumococcal meningitis may occur as a postoperative complication, due to invasion of multidrug-resistant *S. pneumoniae* strains from the nasopharynx after simultaneous osteotomy of the cranium and facial bone in intracraniofacial surgery [43]. Similarly, other nerves of the head may also be important targets for infections, since pneumococcal meningitis is more likely in patients who received cochlear implantation through the surgical insertion technique in proximity to the auditory nerve in the inner ear (cochlea). Occasionally, in the presence of acute otitis media, it is possible that *S. pneumoniae* can reach the CNS via the auditory nerve [44].

In summary, our data offer novel evidence that SCs could be essential for pneumococcal cells to escape phagocytosis and killing by innate immune cells. On the other hand, the results also support the idea of SCs as immunocompetent cells of the PNS that can mediate an efficient immune response against pathogens via MR.

## Competing interests

The authors declare that they have no competing interests.

## Authors’ contributions

HM-R and WB-d-C conceived of the study. HM-R and AFB performed all experiments, except the isolation of the primary Schwann cell cultures. VTR-R and AC-R performed the primary Schwann cell cultures and the infection protocols. HM-R, AFB and LA participated in analyzing the data. HM-R, SA, VTR-R, LMT and WB-d-C participated in designing the study and wrote the final version of the manuscript. LMT and WB-d-C participated in the design and coordination and helped to draft the manuscript. All authors read and approved the final manuscript.

## References

[B1] Thornton JA Durick-Eder K Tuomanen EI Pneumococcal pathogenesis: "innate invasion" yet organ-specific damage J Mol Med 2010 88 103 107 20162252 10.1007/s00109-009-0578-5 PMC2864529

[B2] van Ginkel FW McGhee JR Watt JW Campos-Torres A Parish LA Briles DE Pneumococcal carriage results in ganglioside-mediated olfactory tissue infection Proc Natl Acad Sci U S A 2003 100 14363 1436 14610280 10.1073/pnas.2235844100 PMC283597

[B3] Macedo-Ramos H Campos FS Carvalho LA Ramos IB Teixeira LM De Souza W Cavalcante LA Baetas-da-Cruz W Olfactory ensheathing cells as putative host cells for *Streptococcus pneumoniae*: evidence of bacterial invasion via mannose receptor-mediated endocytosis Neurosci Res 2011 69 308 313 21192991 10.1016/j.neures.2010.12.015

[B4] Herbert RP Harris J Chong KP Chapman J West AK Chuah MI Cytokines and olfactory bulb microglia in response to bacterial challenge in the compromised primary olfactory pathway J Neuroinflammation 2012 9 109 22642871 10.1186/1742-2094-9-109 PMC3411416

[B5] Panni P Ferguson IA Beacham I Mackay-Sim A Ekberg JA St John JA Phagocytosis of bacteria by olfactory ensheathing cells and Schwann cells Neurosci Lett 2013 539 65 70 23415759 10.1016/j.neulet.2013.01.052

[B6] Lisak RP Skundric D Bealmear B Ragheb S The role of cytokines in Schwann cell damage, protection, and repair J Infect Dis 1997 176 173 179 10.1086/513788 9396706

[B7] Baetas-da-Cruz W Alves L Pessolani MC Barbosa HS Regnier-Vigouroux A Corte-Real S Cavalcante LA Schwann cells express the macrophage mannose receptor and MHC class II. Do they have a role in antigen presentation? J Peripher Nerv Syst 2009 14 84 92 19691530 10.1111/j.1529-8027.2009.00217.x

[B8] Goethals S Ydens E Timmerman V Janssens S Toll-like receptor expression in the peripheral nerve Glia 2010 58 1701 1709 20578041 10.1002/glia.21041

[B9] Mattos KA Oliveira VG D'Avila H Rodrigues LS Pinheiro RO Sarno EN Pessolani MC Bozza PT TLR6-driven lipid droplets in *Mycobacterium leprae*-infected Schwann cells: immunoinflammatory platforms associated with bacterial persistence J Immunol 2011 187 2548 2558 21813774 10.4049/jimmunol.1101344

[B10] Medzhitov R Janeway CAJ Innate immunity: The virtues of a nonclonal system of recognition Cell 1997 91 295 298 9363937 10.1016/s0092-8674(00)80412-2

[B11] Varki A Since there are PAMPs and DAMPs, there must be SAMPs? Glycan “self-associated molecular patterns” dampen innate immunity, but pathogens can mimic them Glycobiology 2011 21 1121 1124 21932452 10.1093/glycob/cwr087 PMC3150115

[B12] Martinez-Pomares L The mannose receptor J Leukoc Biol 2012 92 1177 1186 22966131 10.1189/jlb.0512231

[B13] Zamze S Martinez-Pomares L Jones H Taylor PR Stillion RJ Gordon S Wong SY Recognition of bacterial capsular polysaccharides and lipopolysaccharides by the macrophage mannose receptor J Biol Chem 2002 277 41613 41623 12196537 10.1074/jbc.M207057200

[B14] Linehan SA Martínez-Pomares L Stahl PD Gordon S Mannose receptor and its putative ligands in normal murine lymphoid and non-lymphoid organs. In situ expression of mannose receptor by selected macrophages, endothelial cells, perivascular microglia and mesangial cells, but not dendritic cells J Exp Med 1999 189 1961 1972 10377192 10.1084/jem.189.12.1961 PMC2192961

[B15] Hashino M Tachibana M Shimizu T Watarai M Mannose receptor, C type 1 contributes to bacterial uptake by placental trophoblast giant cells FEMS Immunol Med Microbiol 2012 66 427 435 23163874 10.1111/1574-695X.12009

[B16] Régnier-Vigouroux A The mannose receptor in the brain Int Rev Cytol 2003 226 321 342 12921240 10.1016/s0074-7696(03)01006-4

[B17] Giraldi-Guimarães A de Freitas HT de BP C Macedo-Ramos H Mendez-Otero R Cavalcante LA Baetas-da-Cruz W Bone marrow mononuclear cells and mannose receptor expression in focal cortical ischemia Brain Res 2012 1452 173 184 22459039 10.1016/j.brainres.2012.03.002

[B18] Carvalho LA Nobrega AF Soares IDP Carvalho SL Allodi S Baetas-da-Cruz W Cavalcante LA The mannose receptor is expressed by olfactory ensheathing cells in the rat olfactory bulb J Neurosci Res 2013 91 1572 1580 24105692 10.1002/jnr.23285

[B19] Burudi EME Régnier-Vigouroux A Regional and cellular expression of the mannose receptor in the post-natal developing mouse brain Cell Tissue Res 2001 303 307 317 11320646 10.1007/s004410000311

[B20] Baetas-da-Cruz W Alves L Guimaraes EV Santos-Silva A Pessolani MC Barbosa HS Corte-Real S Cavalcante LA Efficient uptake of mannosylated proteins by a human Schwann cell line Histol Histopathol 2009 24 1029 1034 19554510 10.14670/HH-24.1029

[B21] Baetas-da-Cruz W Castro P Guimarães EV Koatz VL Corte-Real S Cavalcante LA Increase in nuclear translocation of nuclear transcription factor-kappaB following infection of a human Schwann cell line with *Leishmania amazonensis* Br J Dermatol 2008 158 631 633 18070200 10.1111/j.1365-2133.2007.08368.x

[B22] Baetas-da-Cruz W Corte-Real S Cavalcante LA Schwann cells as putative safe host cells for *Leishmania amazonensis* Int J Infect Dis 2009 13 e323 e324 19157949 10.1016/j.ijid.2008.11.008

[B23] Morrissey TK Kleitman N Bunge RP Isolation and functional characterization of Schwann cells derived from adult peripheral nerve J Neurosci 1991 11 2433 2442 1869923 10.1523/JNEUROSCI.11-08-02433.1991 PMC6575499

[B24] Ryan JJ Klein KA Neuberger TJ Leftwich JA Westin EH Kauma S Fletcher JA DeVries GH Huff TF Role for the stem cell factor/KIT complex in Schwann cell neoplasia and mast cell proliferation associated with neurofibromatosis J Neurosci Res 1994 37 415 432 7513766 10.1002/jnr.490370314

[B25] Donato R S100: a multigenic family of calcium-modulated proteins of the EF-hand type with intracellular and extracellular functional roles Int J Biochem Cell Biol 2001 33 637 668 11390274 10.1016/s1357-2725(01)00046-2

[B26] Zettler EW Scheibe RM Dias CAG Santafé P Santos DS Moreira JS Fritscher CC Determination of penicillin resistance in *Streptococcus pneumoniae* isolates from southern Brazil by PCR Int J Infect Dis 2006 10 110 115 16310395 10.1016/j.ijid.2005.04.005

[B27] Alves L de Mendonça LL da Silva ME Carvalho L Holy J Sarno EN Pessolani MCV Barker LP *Mycobacterium leprae* infection of human Schwann cells depends on selective host kinases and pathogen-modulated endocytic pathways FEMS Microbiol Lett 2004 238 429 437 15358430 10.1016/j.femsle.2004.08.007

[B28] Roth BL Poot M Yue ST Millard PJ Bacterial viability and antibiotic susceptibility testing with SYTOX green nucleic acid stain Appl Environ Microbiol 1997 63 2421 2431 9172364 10.1128/aem.63.6.2421-2431.1997 PMC168536

[B29] Marzolo MP von Bernhardi R Inestrosa NC Mannose receptor is present in a functional state in rat microglial cells J Neurosci Res 1999 58 387 395 10518112

[B30] Zimmer H Riese S Régnier-Vigouroux A Functional characterization of mannose receptor expressed by immunocompetent mouse microglia Glia 2003 42 89 100 12594740 10.1002/glia.10196

[B31] Vincent AJ Choi-Lundberg DL Harris JA West AK Chuah MI Bacteria and PAMPs activate nuclear factor kappa B and Gro production in a subset of olfactory ensheathing cells and astrocytes but not in Schwann cells Glia 2007 55 905 916 17427933 10.1002/glia.20512

[B32] Ribes S Ebert S Regen T Czesnik D Scheffel J Zeug A Bunkowski S Eiffert H Hanisch UK Hammerschmidt S Nau R Fibronectin stimulates Escherichia coli phagocytosis by microglial cells Glia 2010 58 367 376 19780198 10.1002/glia.20929

[B33] Ribes S Ebert S Regen T Agarwal A Tauber SC Czesnik D Spreer A Bunkowski S Eiffert H Hanisch UK Hammerschmidt S Nau R Toll-like receptor stimulation enhances phagocytosis and intracellular killing of nonencapsulated and encapsulated Streptococcus pneumoniae by murine microglia Infect Immun 2010 78 865 871 19933834 10.1128/IAI.01110-09 PMC2812218

[B34] Iovino F Orihuela CJ Moorlag HE Molema G Bijlsma JJ Interactions between blood-borne Streptococcus pneumoniae and the blood–brain barrier preceding meningitis PLoS One 2013 16 e68408 23874613 10.1371/journal.pone.0068408 PMC3713044

[B35] Ydens E Lornet G Smits V Goethals S Timmerman V Janssens S The neuroinflammatory role of Schwann cells in disease Neurobiol Dis 2013 55 95 103 23523637 10.1016/j.nbd.2013.03.005

[B36] Oliveira RB Ochoa MT Sieling PA Rea TH Rambukkana A Sarno EN Modlin RL Expression of Toll-like receptor 2 on human Schwann cells: a mechanism of nerve damage in leprosy Infect Immun 2003 71 1427 1433 12595460 10.1128/IAI.71.3.1427-1433.2003 PMC148832

[B37] Guiral S Mitchell TJ Martin B Claverys JP Competence-programmed predation of noncompetent cells in the human pathogen *Streptococcus pneumoniae*: genetic requirements Proc Natl Acad Sci U S A 2005 102 8710 8715 15928084 10.1073/pnas.0500879102 PMC1150823

[B38] Claverys JP Havarstein LS Cannibalism and fratricide: mechanisms and raisons d'etre Nat Rev Microbiol 2007 5 219 229 17277796 10.1038/nrmicro1613

[B39] Pérez-Dorado I González A Morales M Sanles R Striker W Vollmer W Mobashery S García JL Martínez-Ripoll M García P Hermoso JA Insights into pneumococcal fratricide from the crystal structures of the modular killing factor LytC Nat Struct Mol Biol 2010 17 576 581 20400948 10.1038/nsmb.1817 PMC6902435

[B40] Tong HH Weiser JN James MA DeMaria TF Effect of influenza A virus infection on nasopharyngeal colonization and otitis media induced by transparent or opaque phenotype variants of *Streptococcus pneumoniae* in the chinchilla model Infect Immun 2001 69 602 606 11119562 10.1128/IAI.69.1.602-606.2001 PMC97928

[B41] Jonsson S Musher DM Chapman A Goree A Lawrence EC Phagocytosis and killing of common bacterial pathogens of the lung by human alveolar macrophages J Infect Dis 1985 152 4 13 3874252 10.1093/infdis/152.1.4

[B42] Noske N Kämmerer U Rohde M Hammerschmidt S Pneumococcal interaction with human dendritic cells: phagocytosis, survival, and induced adaptive immune response are manipulated by PavA J Immunol 2009 183 1952 1963 19570831 10.4049/jimmunol.0804383

[B43] Watanabe Y Akizuki T Prevention and treatment of penicillin-resistant *Streptococcus pneumoniae* meningitis after intracraniofacial surgery with distraction osteogenesis J Craniofac Surg 2008 19 1542 1548 19098547 10.1097/SCS.0b013e31818eece4

[B44] Wei BP Robins-Browne RM Shepherd RK Clark GM O'Leary SJ Can we prevent cochlear implant recipients from developing pneumococcal meningitis? Clin Infect Dis 2008 46 e1 e7 18171202 10.1086/524083

